# Supplementary figures and images for: Experience-Dependent Intrinsic Plasticity in Layer IV of Barrel Cortex at Whisking Onset
Source: eNeuro. 2025 Aug 5;12(8):ENEURO.0252-25.2025. doi: 10.1523/ENEURO.0252-25.2025 (PMC12364433; doi:10.1523/ENEURO.0252-25.2025)

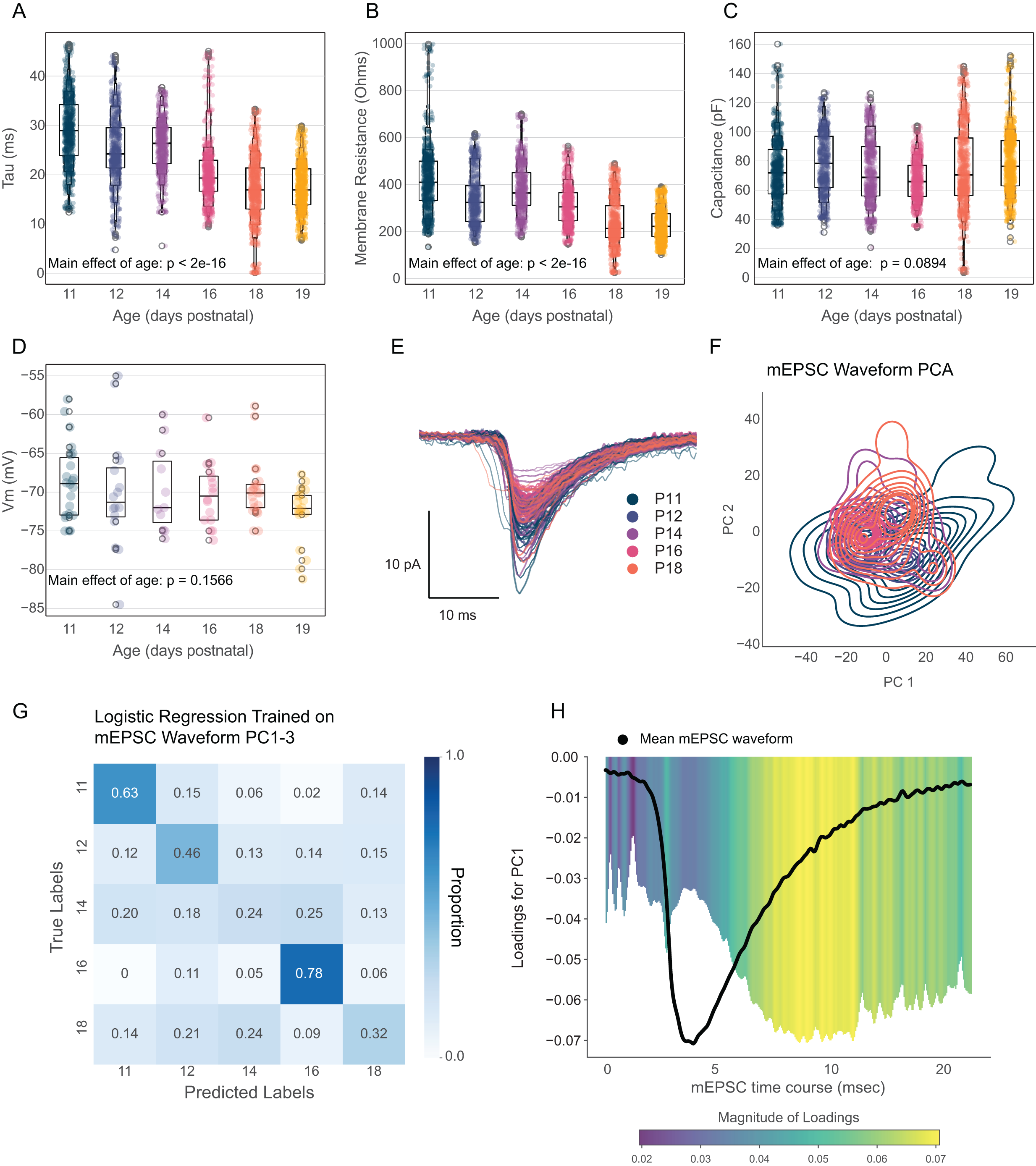

Supplement: Figure 2-1 — Passive membrane properties and mEPSC waveform analysis of layer IV excitatory neurons in the barrel cortex. A-D) Passive membrane properties of layer IV excitatory neurons as a function of age: A) membrane time constant (tau), B) input resistance, C) membrane capacitance, and D) resting membrane potential. E) Mean mEPSC waveforms from layer IV excitatory neurons across development (P11-P18). F) Kernel density estimate plot showing the the first two principal components of PCA run on the mean waveforms shown in E. Color indicates age as in E. G) Confusion matrix for a logistic regression model trained to predict age based on the first three principal components of mEPSC waveforms. H) Loadings for the first principal component (PC1), illustrating. Download Figure 2-1, TIF file. [file eneuro-12-ENEURO.0252-25.2025-s001.tif]

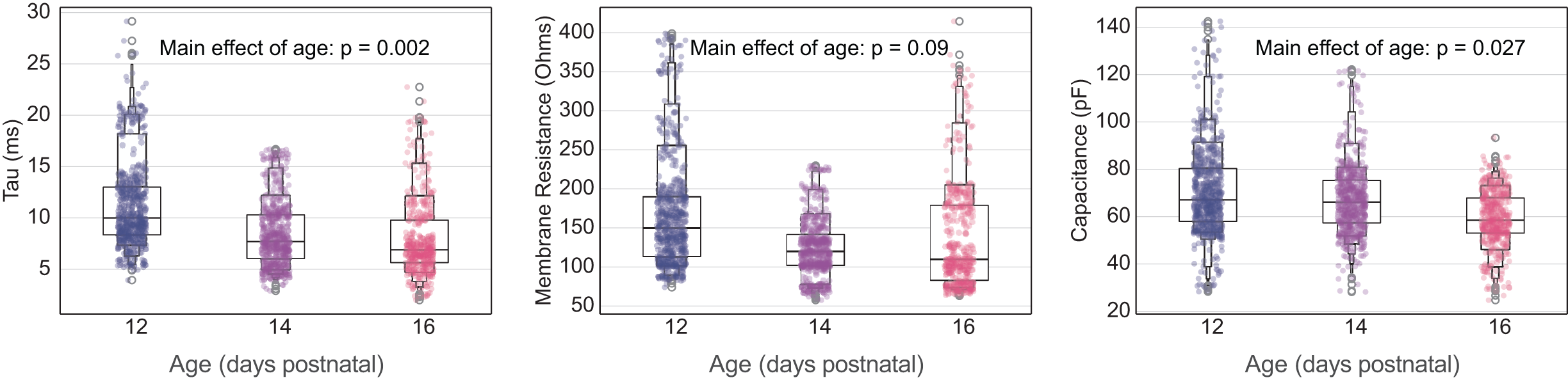

Supplement: Figure 3-1 — Passive membrane properties of putative parvalbumin (PV) and somatostatin (SST) interneurons in layer IV of the barrel cortex. Passive membrane properties of putative PV and SST interneurons as a function of age (P12, P14, and P16): (Left) membrane time constant (tau), (Center) input resistance, and (Right) membrane capacitance. Download Figure 3-1, TIF file. [file eneuro-12-ENEURO.0252-25.2025-s002.tif]

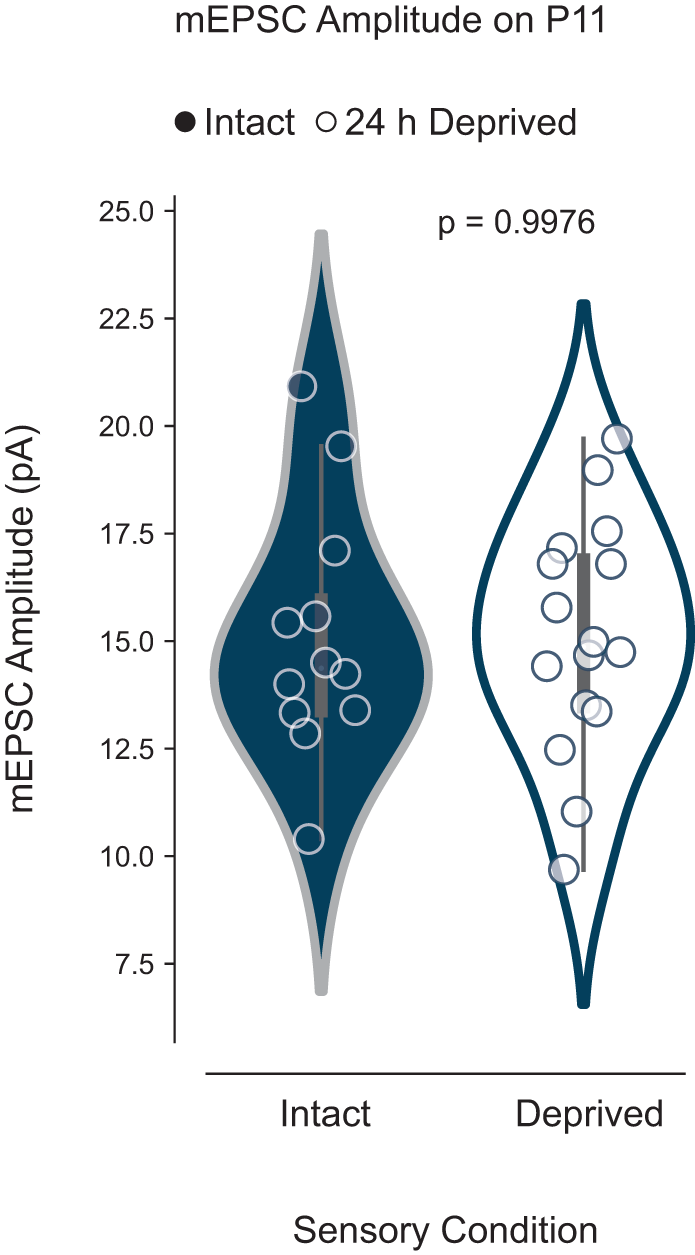

Supplement: Figure 6-1 — Whisker deprivation does not induce homeostatic synaptic scaling in layer IV excitatory neurons at P11. Comparison of mEPSC amplitudes between intact and deprived hemispheres in layer IV excitatory neurons at P11, 24 h after the onset of unilateral whisker deprivation. No significant difference was observed between the two conditions (p = 0.9976), indicating an absence of homeostatic synaptic scaling at this age and time point. Download Figure 6-1, TIF file. [file eneuro-12-ENEURO.0252-25.2025-s003.tif]
